# Two-year survival after scheduled extubation in patients with pneumonia or ARDS: a prospective observational study

**DOI:** 10.1186/s12871-024-02603-9

**Published:** 2024-07-10

**Authors:** Xuemin Chai, Mengyi Ma, Wenhui Hu, Linfu Bai, Jun Duan

**Affiliations:** Department of Respiratory and Critical Care Medicine, the First Affiliated Hospital of Chongqing Medical University, Youyi Road 1, Yuzhong District, Chongqing, 400016 P. R. China

**Keywords:** Comorbidity, Disease severity, Cough strength, Extubation failure, Survival

## Abstract

**Purpose:**

To report two-year survival after scheduled extubation in patients with pneumonia or acute respiratory distress syndrome (ARDS).

**Methods:**

This was a prospective observational study performed in a respiratory ICU of a teaching hospital. Pneumonia or ARDS patients who successfully completed a spontaneous breathing trial were enrolled. Data were collected before extubation. Patients were followed up to two years by phone every 3 months.

**Results:**

A total of 230 patients were enrolled in final analysis. One-, 3-, 6-, 12-, and 24-month survival was 77.4%, 63.8%, 61.3%, 57.8%, and 47.8%, respectively. Cox regression shows that Charlson comorbidity index (hazard ratio: 1.20, 95% confidence interval: 1.10–1.32), APACHE II score before extubation (1.11, 1.05–1.17), cough peak flow before extubation (0.993, 0.986–0.999), and extubation failure (3.96, 2.51–6.24) were associated with two-year mortality. To predict death within two years, the area under the curve of receiver operating characteristic was 0.79 tested by Charlson comorbidity index, 0.75 tested by APACHE II score, and 0.75 tested by cough peak flow. Two-year survival was 31% and 77% in patients with Charlson comorbidity index ≥ 1 and < 1, 28% and 62% in patients with APACHE II score ≥ 12 and < 12, and 64% and 17% in patients with cough peak flow > 58 and ≤ 58 L/min, respectively.

**Conclusions:**

Comorbidity, disease severity, weak cough and extubation failure were associated with increased two-year mortality in pneumonia or ARDS patients who experienced scheduled extubation. It provides objective information to caregivers to improve decision-making process during hospitalization and post discharge.

## Introduction

Mechanical ventilation is commonly used in patients with acute respiratory failure. When the respiratory failure has been reversed, ventilator weaning should be considered. However, if the patient is unable to tolerate spontaneous breathing, re-intubation will happen. On the other hand, if the patient is able to tolerate spontaneous breathing and is still undergoing mechanical ventilation, it will increase the risk of ventilator-associated pneumonia [1, 2]. How to balance premature and delayed ventilator weaning is important. Spontaneous breathing trial (SBT) has been introduced to manage ventilator weaning, which considers both sides [3–5].

After a successful SBT, endotracheal intubation is recommended to be removed. The hospital survival is 80% to 90% in these patients [6, 7]. And one-year survival is 41% to 76% [8, 9]. In mixed population, positive fluid balance, advanced age, frailty, presence of COPD, and low hemoglobin are associated with long-term mortality [10–13]. However, the long-term outcome in patients with pneumonia or acute respiratory distress syndrome (ARDS) is lacking. Here, we aimed to report two-year survival after a successful SBT in patients with pneumonia or ARDS, and further explore the risk factors associated with death within two years.

## Methods

We undertook a prospective observational study in a respiratory intensive care unit (ICU) of a teaching hospital from February 2011 to November 2019. The last patient was followed up to November 2021. Patients who passed a SBT and readiness for extubation were candidates for this study. The inclusion criteria were age more than 18 years and diagnosis as pneumonia or ARDS. The exclusion criteria were presence of tracheotomy, refusal of participation, and lost to follow up. The study protocol was approved by the ethics committee and institutional review board of the First Affiliated Hospital of Chongqing Medical University (No: 2011/05/13 and 2016/11/10). The informed consent was waived due to the observational nature.

Diagnosis of pneumonia was based on current guidelines: new or increasing pulmonary infiltrate in chest x-ray or computerized tomography along with clinical findings suggesting infection, such as new onset of fever, cough, chest pain, purulent sputum, shortness of breath, leukocytosis, decline in oxygenation, and so on [14, 15]. Diagnosis of ARDS was also based on current guideline: 1) within 1 week of a clinical insult or the presence of new (≤ 7 days) or worsening respiratory symptoms; 2) bilateral opacities on chest X-ray or computerized tomography not fully explained by effusions, lobar or lung collapse, or nodules; 3) respiratory failure not fully explained by cardiac failure or fluid overload; and 4) presence of acute hypoxemic respiratory failure with PaO_2_/FiO_2_ less than 300 mm Hg [16].

We managed the patients according to current guidelines and our hospital’s protocols [17–19]. The propofol, dexmedetomidine, midazolam, fentanyl and morphine were used to manage sedation and analgesia. It was titrated to achieve Richmond agitation sedation scale of -2 to 0 or Ramsay score of 3 to 4. Exercise and mobilization at beside were performed as early as possible to speed ventilator weaning. Elevation of the head of the bed at 30 to 45℃, subglottic suctioning, hand hygiene, and oral care were used to prevent ventilator-associated pneumonia. Enteral nutrition was given according to the energy consumption. If the enteral nutrition was inadequate, parenteral nutrition was given. In patients with septic shock, fluid resuscitation was used. However, fluid should be limited in patients with excessive extravascular lung water.

Every morning, respiratory therapists and physicians screened the patients to identify the candidates who would initiate the weaning process. The criteria for initiation of weaning process were as follows: improvement or resolution of the underlying cause of acute respiratory failure, improvement of oxygenation (PaO_2_/FiO_2_ > 200 mm Hg), reduction of ventilator support (FiO_2_ ≤ 0.5 and positive-end expiratory pressure < 8 cmH_2_O), temperature < 38 °C, no acidosis, no tachypnea (breathing frequency < 30 breaths/min), no tachycardia (heart rate < 120 beats/min), and no hypotension or hypertension (systolic blood pressure between 90 and 180 mm Hg).

If the patients reached the criteria for initiation of the weaning process, a 30- to 120-min SBT was performed. Low level of pressure support ventilation (6–8 cm H_2_O) was used to test the patients’ ability to breathe spontaneously. If the SBT failed, previous ventilation parameters were used again and weaning attempt was assessed next day. The criteria for the failure of SBT were as follows: rapid shallow breathing index (f/Vt) > 105, respiratory rate > 35 breaths/min, SpO_2_ < 90% with a FiO_2_ > 0.5, systolic blood pressure > 180 or < 90 mm Hg, heart rate > 140 or < 50 beats/min, pH < 7.35, diminishing consciousness or diaphoresis, and clinical signs indicating respiratory muscle fatigue, labored breathing, or both [20].

If a patient successful completed a SBT, extubation was performed. Before extubation, cough peak flow was assessed. Patients were coached to cough through the endotracheal tube as much effort as possible to measure the cough peak flow by an external flowmeter or a built-in ventilator flowmeter [21, 22]. After extubation, noninvasive ventilation (NIV) or high flow nasal cannula (HFNC) was immediately used in patients at high risk for extubation failure. The risk factors included age ≥ 65 years, body mass index ≥ 30 kg/m^2^, two or more comorbidities, weak cough, presence of chronic cardiopulmonary disease, mechanical ventilation ≥ 7 days, and difficult or prolong weaning [23–25]. All the patients with a scheduled extubation were followed up by phone every 3 months to two years after extubation.

### Statistical analysis

The SPSS version 17.0 (SPSS, Chicago, IL, USA) was used to analyze the data. Qualitative and categorical variables were reported as numbers and percentages. The differences between groups were analyzed by χ2 test or Fisher’s exact test as appropriate. Normally distributed continuous variables were reported as mean values and standard deviations, and differences between groups were analyzed by independent sample t test. Abnormally distributed continuous variables were reported as median values and interquartile ranges, and differences between groups were analyzed by Mann–Whitney U test. Cox regression was used to analyze the hazard ratio (HR) of two-year mortality. The cumulative two-year survival probability was created by Kaplan–Meier curves. Differences between two curves were analyzed by log-rank test. The area under the curve of receiver operating characteristic (AUC) was used to analyze the predictive power of death at two years after extubation. The optimal cutoff value was determined at the maximum Youden index [26]. Statistical significance was determined if a *p* value was less than 0.05.

## Results

We initially enrolled 234 cases in current study. However, 4 of them were lost to follow up. Therefore, only 230 patients were enrolled in the final analysis. In the total cohort, the mean age was 63 years, the median duration of mechanical ventilation before extubation was 6 days, and the mean APACHE II score before extubation was 12 (Table 1). Most of the cases were males (71%). Simple weaning accounted for 73%. Two out of five patients used NIV or HFNC as a preventive strategy. Only 14% of patients experienced extubation failure.

**Table 1:** Baseline data

	Total cohort *N* = 230	Survivor *N* = 110	Nonsurvivor *N* = 120	*p*
Age, years	63 ± 19	55 ± 20	70 ± 16	< 0.01
Male	164 (71%)	75 (68%)	89 (74%)	0.38
Charlson comorbidity index	1 (0–2)	0 (0–1)	2 (1–3)	< 0.01
Weaning category				
Simple weaning	167 (73%)	88 (80%)	79 (66%)	0.05
Difficult weaning	47 (20%)	16 (14%)	31 (26%)	
Prolonged weaning	16 (7%)	6 (6%)	10 (8%)	
Variables collected before extubation				
GCS	14.6 ± 1.3	14.9 ± 0.4	14.4 ± 1.8	< 0.01
Duration of MV before extubation, d	6 (4–9)	6 (4–9)	7 (3–9)	0.87
APACHE II score	12 ± 4	11 ± 3	14 ± 4	< 0.01
Hemoglobin, g/dl	10.2 ± 2.1	10.6 ± 2.1	9.9 ± 2.2	0.01
Albumin, g/L	29 ± 5	30 ± 5	28 ± 6	< 0.01
Respiratory rate, breaths/min	23 ± 6	22 ± 6	23 ± 6	0.11
Rapid shallow breathing index	54 ± 25	50 ± 27	58 ± 23	0.02
Heart rate, beats/min	95 ± 16	95 ± 15	95 ± 16	0.95
Systolic blood pressure, mmHg	133 ± 25	133 ± 26	133 ± 24	0.87
Diastolic blood pressure, mmHg	73 ± 14	75 ± 14	71 ± 13	0.05
PH	7.44 ± 0.05	7.45 ± 0.05	7.44 ± 0.06	0.52
PaCO_2,_ mmHg	41 ± 11	41 ± 10	41 ± 12	0.91
PaO_2_/FiO_2,_ mmHg	254 ± 88	254 ± 90	254 ± 86	0.97
Cough peak flow, L/min^a^	82 ± 42	101 ± 42	68 ± 36	< 0.01
Preventive use of NIV or HFNC	94 (41%)	39 (36%)	55 (46%)	0.14
Extubation failure at 72 h	32 (14%)	3 (3%)	29 (24%)	< 0.01
Duration of hospital stay, days	22 (14–33)	20 (14–29)	24 (14 -41)	0.06
Duration of ICU stay, days	12 (8–18)	11 (7–17)	14 (9–21)	< 0.01
Duration of hospital stay after extubation, days	12 (6–19)	11 (7–19)	12 (5–21)	0.92
Duration of ICU stay after extubation, days	5 (2–9)	4 (2–6)	6 (2–14)	< 0.01

*MV* mechanical ventilation, *NIV* noninvasive ventilation, *HFNC* high-flow nasal cannula, *ICU* intensive care unit

^a^Two cases were missed

**Fig. 1:**
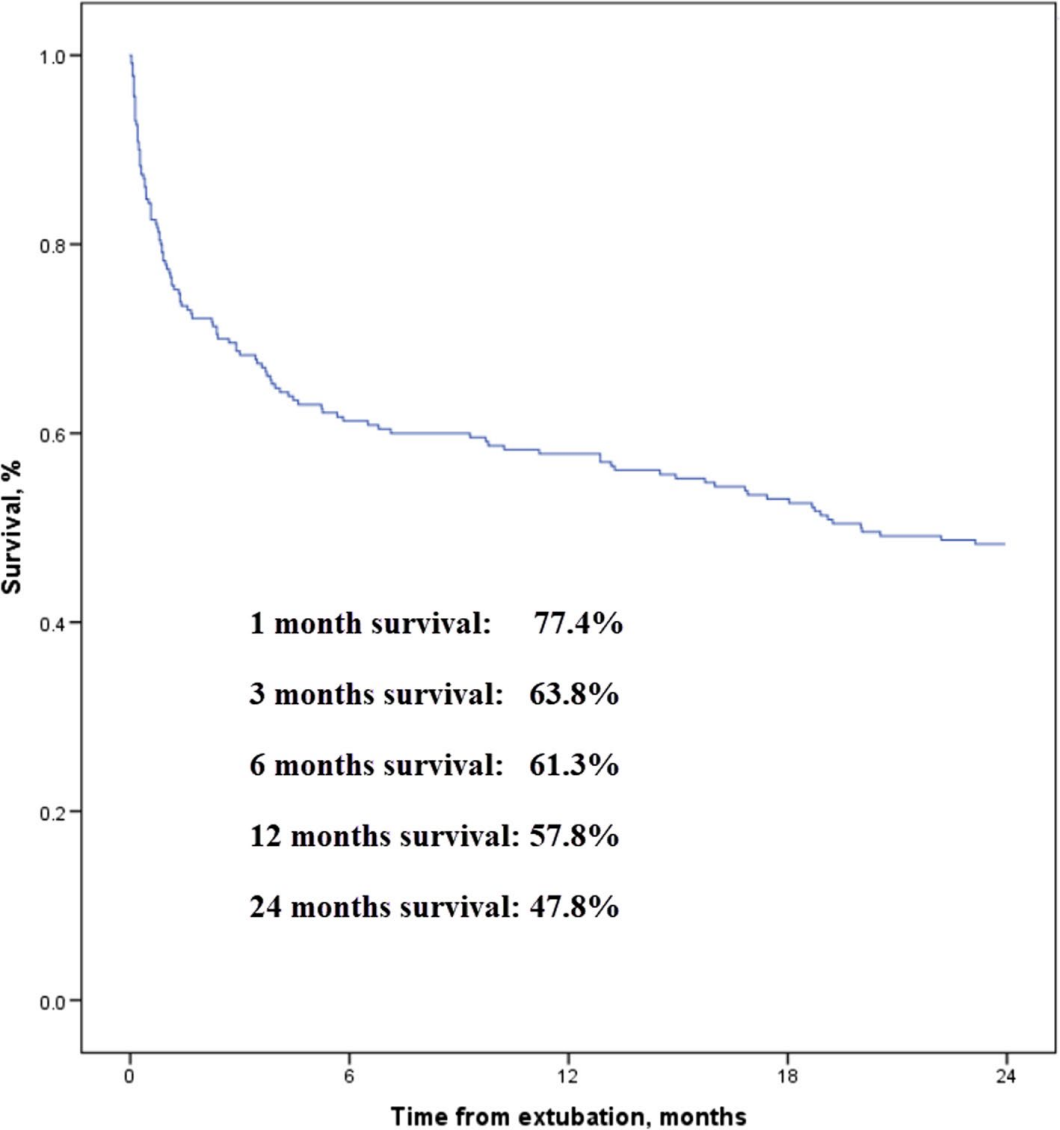
Two-year survival in total cohort

One-month survival was 77.4% (Fig. 1). It decreased to 63.8% at 3 months, 61.3% at 6 months, 57.8% at 12 months and 47.8% at 24 months. The nonsurvivors were older than survivors (mean 70 vs. 55 years, *p* < 0.01). They also had higher Charlson comorbidity index (median 2 vs. 0, *p* < 0.01), higher APACHE II score (mean 14 vs. 11, *p* < 0.01), higher proportion of extubation failure (24% vs. 3%, *p* < 0.01), lower albumin (mean 28 vs. 30 g/L, *p* < 0.01), and lower cough peak flow (mean 68 vs. 101 L/min, *p* < 0.01) than survivors. There were no differences in arterial blood gas tests between survivors and nonsurvivors.

The cox regression shows that Charlson comorbidity index, APACHE II score before extubation, cough peak flow and extubation failure were associated with two-year mortality (Table 2). The HR of two-year mortality was 1.20 (95% confidence interval [CI]: 1.10–1.32) for one unit increase of Charlson comorbidity index, 1.11 (95%CI: 1.05–1.17) for one unit increase of APACHE II score, 0.993 (95%CI: 0.986–0.999) for one unit increase of cough peak flow, and 3.96 (95%CI: 2.51–6.24) for extubation failure.

**Table 2:** Cox regression analysis for two-year mortality

	HR (95% CI)	*p*
Charlson comorbidity index	1.20 (1.10–1.32)	< 0.01
APACHE II score before extubation	1.11 (1.05–1.17)	< 0.01
Cough peak flow	0.993 (0.986–0.999)	0.03
Extubation failure	3.96 (2.51–6.24)	< 0.01

The age, sex, Charlson comorbidity index, weaning category, extubation failure, duration of mechanical ventilation before extubation, APACHE II score, mental status, hemoglobin, albumin, cough peak flow, prophylactic use of noninvasive ventilation or high-flow nasal cannula, vital signs, and arterial blood gas tests were entered into the cox regression model

*HR* hazard ratio, *CI* confidence interval

**Fig. 2:**
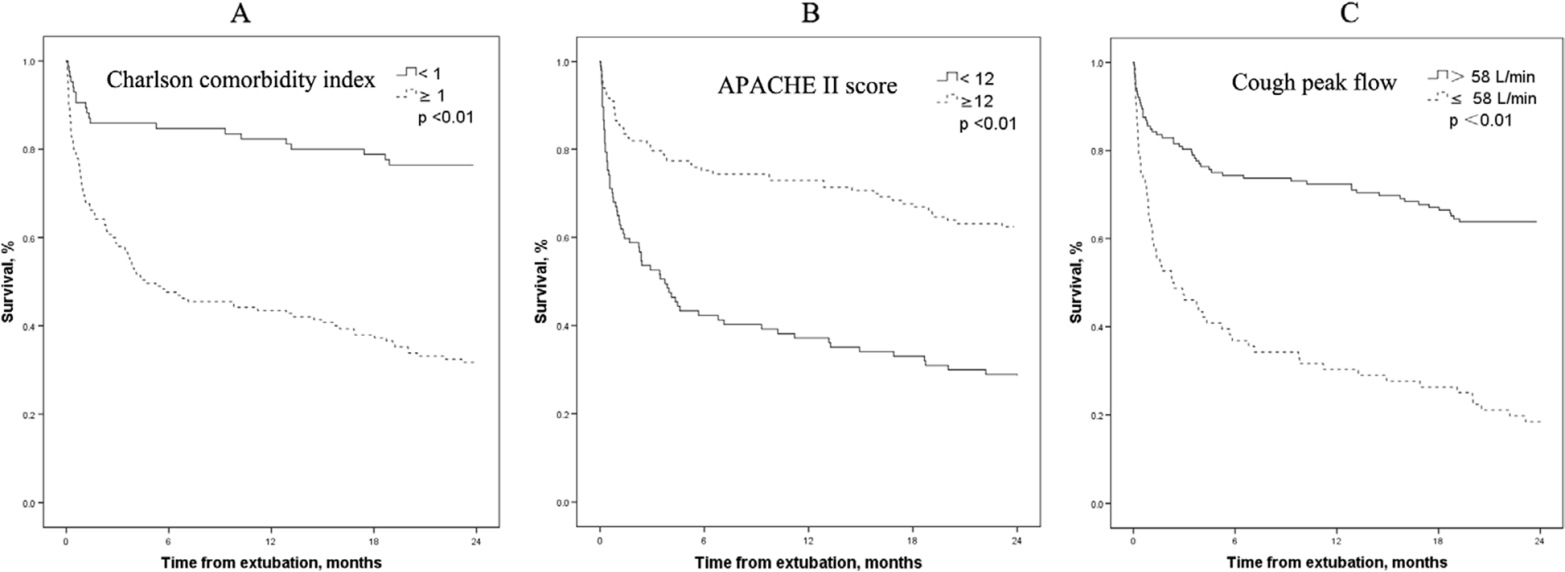
Two-year survival in patients with low and high probability of death classified by Charlson comorbidity index, APACHE II score, and cough peak flow

To predict death within two years after extubation, the AUC was 0.79 tested by Charlson comorbidity index, 0.75 tested by APACHE II score, and 0.75 tested by cough peak flow (Table 3). The optimal cutoff values were 1 point for Charlson comorbidity index, 12 points for APACHE II score, and 58 L/min for cough peak flow. Two-year survival was 31% (45/145) and 77% (65/85) in patients with Charlson comorbidity index ≥ 1 and < 1, 28% (27/97) and 62% (83/133) in patients with APACHE II score ≥ 12 and < 12, and 64% (97/152) and 17% (13/76) in patients with cough peak flow > 58 and ≤ 58 L/min, respectively (Fig. 2).

**Table 3:** Predictive power of two year mortality

	AUC (95%CI)	Cutoff value	Sensitivity	Specificity
Charlson comorbidity index	0.79 (0.71–0.86)	≥ 1	83%	59%
APACHE II score before extubation	0.75 (0.69–0.81)	≥ 12	75%	66%
Cough peak flow, L/min	0.75 (0.68–0.81)	≤ 58	53%	88%

*AUC* area under the curve of receiver operating characteristic, *CI* confidence interval

## Discussion

Current study shows that half of the pneumonia or ARDS patients with a scheduled extubation after a successful SBT died within two years after extubation. Most of them died within the first 3 months. Charlson comorbidity index, APACHE II score, cough peak flow, and extubation failure were strongly associated with two-year mortality.

The average rate of extubation failure is 15% [27]. In our study, the extubation failure was 14%, which was similar with the average rate. This indicates that the management of mechanical ventilation in our unit was in the medium levels. It is not surprising that extubation failure is associated with increased mortality. However, most of the studies only reported the short-term outcomes, and the management strategies mainly focused on several days after extubation [6, 7, 28, 29]. Our study reported two-year mortality among patients who successfully completed a SBT. Half of the patients died within two years, and most of the death occurred within the first 3 months. These data provide important information for policy makers, physicians, and nurses to manage patients after extubation. More attention should be paid in the first 3 months.

Comorbidity and disease severity are associated with increased mortality in critically ill patients [30, 31]. Our study confirmed these issues. Different with previous studies, we quantitatively described these two variables. We used Charlson comorbidity index and APACHE II score to assess the comorbidity and disease severity accurately, and found that the power to predict death within two years after extubation was high. We further explored these two variables, and determined that 1 point of Charlson comorbidity index and 12 points of APACHE II score were the optimal cutoff values. Beyond the cutoff values, the risk of death significantly increased. These detailed data are helpful when the physicians make the decision of extubation and provide the post-extubation managing strategies.

Weak cough was another risk factor associated with two-year mortality in our study. Cough strength is positively correlated with maximal inspiratory pressure [32]. And the maximal inspiratory pressure is associated with long-term mortality in patients after a scheduled extubation [33]. Therefore, patients with lower maximal inspiratory pressure are more likely to die after extubation. This is one reason for the association between weak cough and increased two-year mortality. In addition, weak cough is associated with aspiration, which increases the risk of pneumonia within one year after hospitalization [34–36]. This is another reason why weak cough is associated with increased two-year mortality.

In addition to the risk factors associated with long-term mortality reported in our study, positive fluid balance, advanced age, frailty, presence of COPD, and low hemoglobin are associated with long-term mortality [10–13]. The nutritional status, mental status, support systems in place for patient care post-discharge, the rate of infection or ventilator-acquired pneumonia is also contributed much to two-year mortality. However, we did not record these risk factors, which may influence our results.

Current study may be limited by the methodology. First, it was performed in a respiratory intensive care unit. The patient population may not represent all patients with pneumonia or ARDS due to regional, demographic, or healthcare delivery differences. The selection bias may affect the generalizability of the findings. Second, we only reported that Charlson comorbidity index, APACHE II score, cough strength and extubation failure were associated with two-year mortality. It’s essential to remember these are not necessarily causal relationships as the observational design. Third, we only recorded the rate of death within two years after extubation. The reasons for death are unclear. It is unable to ascertain whether a direct correlation exists between altered peak cough flow and patient mortality.

## Conclusion

Half of the pneumonia or ARDS patients after a successful SBT died within two years after extubation. Comorbidity, disease severity before extubation, weak cough and extubation failure were associated with two-year mortality. It provides objective information to caregivers to improve decision-making process during hospitalization and after discharge.

## Data Availability

The datasets analyzed during the current study available from the corresponding author on reasonable request.
